# Change in cardiometabolic risk factors in a pilot safety-net plant-based lifestyle medicine program

**DOI:** 10.3389/fnut.2023.1155817

**Published:** 2023-04-20

**Authors:** Stephanie L. Albert, Rachel E. Massar, Lilian Correa, Lorraine Kwok, Shivam Joshi, Sapana Shah, Rebecca Boas, Héctor E. Alcalá, Michelle McMacken

**Affiliations:** ^1^NYU Grossman School of Medicine, New York, NY, United States; ^2^NYC Health + Hospitals, New York, NY, United States; ^3^Veterans Affairs, Orlando, FL, United States; ^4^Department of Behavioral and Community Health, School of Public Health, University of Maryland, College Park, MD, United States; ^5^Program in Oncology, University of Maryland Marlene and Stewart Greenebaum Comprehensive Cancer Center, Baltimore, MD, United States

**Keywords:** plant-based diet, lifestyle medicine, chronic disease, lifestyle modification, cardiovascular risk, outcome evaluation, lifestyle intervention, cardiometabolic risk

## Abstract

**Introduction:**

Interventions emphasizing healthful lifestyle behaviors are proliferating in traditional health care settings, yet there is a paucity of published clinical outcomes, outside of pay-out-of-pocket or employee health programs.

**Methods:**

We assessed weight, hemoglobin A1c (HbA1c), blood pressure, and cholesterol for 173 patients of the Plant-Based Lifestyle Medicine Program piloted in a New York City safety-net hospital. We used Wilcoxon signed-rank tests to assess changes in means, from baseline to six-months, for the full sample and within baseline diagnoses (i.e., overweight or obesity, type 2 diabetes, prediabetes, hypertension, hyperlipidemia). We calculated the percentage of patients with clinically meaningful changes in outcomes for the full sample and within diagnoses.

**Findings:**

The full sample had statistically significant improvements in weight, HbA1c, and diastolic blood pressure. Patients with prediabetes or overweight or obesity experienced significant improvements in weight and those with type 2 diabetes had significant improvements in weight and HbA1c. Patients with hypertension had significant reductions in diastolic blood pressure and weight. Data did not show differences in non-high-density lipoprotein cholesterol (non-HDL-C), but differences in low-density lipoprotein cholesterol (LDL-C) were approaching significance for the full sample and those with hyperlipidemia. The majority of patients achieved clinically meaningful improvements on all outcomes besides systolic blood pressure.

**Conclusion:**

Our study demonstrates that a lifestyle medicine intervention within a traditional, safety-net clinical setting improved biomarkers of cardiometabolic disease. Our findings are limited by small sample sizes. Additional large-scale, rigorous studies are needed to further establish the effectiveness of lifestyle medicine interventions in similar settings.

## Introduction

Chronic diseases such as heart disease, cancer, and type 2 diabetes are among the leading causes of death and disability in the United States (1). Sixty percent of adults live with at least one chronic disease and 40% live with two or more (2). Given increased prescription costs, the need for continuous care, and more frequent health care visits, the management of chronic diseases accounts for 90% of annual health care expenditures--making chronic diseases the leading drivers of healthcare costs in the U.S. (3–5) Health behaviors such as tobacco use, poor nutrition characterized by diets high in sodium and saturated fats and low in fruits and vegetables, lack of physical activity, and insufficient sleep are modifiable risk factors that contribute to the development of chronic disease (6).

There is strong evidence supporting the use of lifestyle interventions to prevent and treat chronic disease. For example, healthful, plant-predominant diets have been associated with significant reductions in the risk of ischemic heart disease, cancer, type 2 diabetes, obesity, and hypertension (7–12). This dietary pattern has also been shown to improve existing conditions such as coronary artery disease, type 2 diabetes, and obesity (12–17). In addition, other lifestyle behaviors including regular physical activity, restorative sleep, and stress management are associated with positive health outcomes (18–20). Leading medical organizations including the American Diabetes Association (21), American Association of Clinical Endocrinology (22), American College of Cardiology (23), American Heart Association (24), American Society for Preventive Cardiology (25), National Lipid Association (26), and the American Cancer Society (27) all recommend lifestyle change, including plant-predominant eating patterns, as first-line therapy.

Lifestyle medicine is a medical specialty that uses therapeutic lifestyle interventions as a primary modality to treat chronic lifestyle-related conditions (28). Trained clinicians deliver evidence-based care focused on encouraging a healthful, plant-predominant eating pattern, regular physical activity, restorative sleep, stress management, avoidance of risky substances, and positive healthy relationships. Lifestyle medicine has proliferated rapidly in recent years. More than 80 major health systems across the United States currently participate in the Health Systems Council for the American College of Lifestyle Medicine, indicating their commitment to integrating lifestyle medicine into their services (29). Lifestyle medicine interventions have been shown to improve cardiometabolic risk factors by lowering weight, blood pressure, fasting lipids, and blood sugar, while reducing angina and cardiac events (13, 30). However, there is a paucity of published data on the clinical impact of lifestyle medicine programs in traditional health care settings, outside of employee health, pay-out-of-pocket, or residential programs. Furthermore, even fewer studies have examined the impact of a lifestyle medicine program within a safety-net clinical setting that serves high-needs populations experiencing disproportionate rates of chronic diseases.

The Plant-Based Lifestyle Medicine (PBLM) Program is a pilot clinical program established in an urban public healthcare system. The program’s goal is to help patients adopt a healthful plant-based eating pattern and other positive behaviors. The evaluation of the pilot program focused primarily on feasibility of implementation and demand for the program (31). We found demand for the program was high, far exceeding capacity, and patients reported joining the program in order to gain control over their lives, reduce their medication burden, and lose weight. The program’s team, overall approach, and resources all were popular among patients despite reported barriers to accessing the program as well as administrative and insurance challenges. In this paper, we build on the implementation findings of the PBLM Program by assessing preliminary data on clinical outcomes including blood sugar, weight, blood pressure, and cholesterol among program patients. Our study is novel because, to our knowledge, the pilot PBLM Program is the first lifestyle medicine program operating within a traditional safety-net healthcare system to publish clinical outcomes data.

## Methods

The primary goal of the pilot PBLM Program evaluation was to assess the feasibility of implementing the program and the demand for its services. A secondary goal was to assess preliminary data related to clinical outcomes. The study uses a pre/post single sample design. Approval for this study was obtained from both the New York University (NYU) Grossman School of Medicine Institutional Review Board as well as the Office of Research and Administration for Implementation at NYC Health + Hospitals/Bellevue.

### Intervention

A detailed description of the 1-year pilot of the PBLM Program has been published previously (31). Briefly, the PBLM Program at NYC Health + Hospitals/Bellevue, a large public hospital in New York City, was designed to provide structured, interdisciplinary support for lifestyle changes to improve patients’ cardiometabolic health. Adults with excess weight (i.e., BMI ≥ 25), type 2 diabetes, prediabetes, hypertension, atherosclerotic heart disease, and/or hyperlipidemia were eligible to participate. Individuals learned of the program through earned media (e.g., TV news, newspapers), social media, announcements from the office of the Brooklyn Borough President, and limited health care provider referrals from within Bellevue. Thus, most patients self-selected into the program and joined by adding their name to a waitlist. Enrollment in the program was on a first-come, first-serve basis. The program encouraged patients to transition to a healthful plant-based diet, improve sleep health, increase physical activity, improve stress management, foster positive social connections, and avoid substance use. Patients met individually with clinicians who have expertise in plant-based nutrition and lifestyle medicine, including a physician, a registered dietitian, and a health coach, to set goals and monitor progress. Group classes supplemented one-on-one visits, emphasizing education, skills-building, action planning, and peer-to-peer support. In addition, an exercise trainer offered classes focused on aerobic and strength training. Resources available to all patients included a plant-based diet starter guide, cookbook(s), HealthBucks (coupons that can be used to purchase fresh fruits and vegetables at all New York City farmer’s markets), a Healthy Savings Card for grocery store discounts on fresh produce, and access to a private Facebook group for social support, advice, and recipe sharing. The frequency and duration of program engagement for each individual was determined jointly by the PBLM Program provider team and each of the patients.

### Data sources

Clinical outcomes for all patients of the PBLM Program were analyzed using de-identified electronic health record (EHR) data. In total, 173 individuals had at least one visit with the program between January 16, 2019 and February 15, 2020 and had EHR data. Because data were de-identified, consent was not required. Data were pulled at one time point and contained all recorded values during the study period. Additionally, program staff maintained a separate administrative database to track the following data for all patients: (1) limited demographic data (i.e., age, gender); (2) baseline clinical diagnoses determined by the treating physician (i.e., type 2 diabetes, prediabetes, overweight or obesity, hypertension, heart disease, hyperlipidemia); and (3) medications and their dosages at baseline, three-months, and six-months. The two datasets were merged for analyses using a unique identifier.

### Measures

There were six clinical outcomes of interest in this study: (1) weight (kg); (2) hemoglobin A1c (HbA1c) (%); (3) systolic blood pressure (mm Hg); (4) diastolic blood pressure (mm Hg); (5) non-high-density lipoprotein cholesterol (non-HDL-C) (mg/dl); and (6) low-density lipoprotein cholesterol (LDL-C) (mg/dl).

In order to assess the contribution of behavior change to clinical outcomes, independent of medication changes, we created a stable or reduced medication regimen variable for those with type 2 diabetes, hypertension, and hyperlipidemia diagnoses at baseline. We first calculated the total number of medications prescribed for each participant within diagnoses (e.g., total number of diabetes medications prescribed for someone with type 2 diabetes) at baseline and six-months. Then we coded changes to medications by calculating the difference in the total number of medications from baseline to six-months (+1 for new medication added, −1 for medication removed, 0 for no change in medications). For those with no change in the number of disease-specific medications, we assessed changes in dosage (+1 for increase, −1 for decrease, 0 for no change). We then combined these two factors for a medication and dosage variable where a positive number indicates a net increase (increased medication regimen), a negative number indicates a net decrease (reduced medication regimen), and 0 indicates no net change (stable medication regimen).

In addition, we created variables to represent clinically meaningful change in each of the six clinical outcomes. Clinically meaningful improvement was based on published literature and was defined as follows: ≥3% decrease in weight (32, 33), ≥0.5 percentage point reduction in HbA1c for patients with a baseline diagnosis of type 2 diabetes (34–36), no change or any decrease in HbA1c for patients with a baseline diagnosis of prediabetes (i.e., no progression to type 2 diabetes) (37), ≥3 mm Hg reduction in systolic or diastolic blood pressure (38, 39), and any decrease in non-HDL-C or LDL-C (40, 41). First, we calculated the change from baseline to six-months (positive number indicates an increase, negative number indicates a decrease, zero indicates no change). Then we created a binary variable (yes/no) to indicate whether that change was clinically meaningful for each of the clinical outcomes.

### Analyses

We assessed comparability in demographic characteristics and baseline diagnoses by gender using Chi-squared tests. We conducted analyses to assess changes in patients’ clinical outcomes between baseline and six-months for the full sample and then within diagnosis (i.e., overweight or obesity, type 2 diabetes, prediabetes, hypertension, hyperlipidemia) using only patients that had data at both time points; heart disease was excluded from subgroup analysis due to the small sample size. When analyzing outcomes within the baseline diagnosis subgroups, we assessed change over time only for the most relevant outcomes (e.g., non-HDL-C and LDL-C for patients with hyperlipidemia) in addition to weight because a substantial majority (i.e., ≥ 80%) of patients had a BMI ≥ 25 within each condition. We also assessed change in weight and relevant clinical outcomes for three disease-specific subgroups of patients (i.e., type 2 diabetes, hypertension, hyperlipidemia) with stable or reduced disease-specific medication regimens over the study period. Our goal in reviewing outcomes by stable or reduced medication status was to isolate, to the best possible extent, the effects of lifestyle change alone. Change in medication status was not reviewed for the prediabetes, overweight or obesity, or heart disease subgroups, given that disease-specific medications were not used for prediabetes or overweight or obesity, and the sample size of the heart disease subgroup was too small for analysis. For most patients, baseline values were established at the first appointment. In selected cases where an individual did not have clinical data from their first visit, we used lab results that were no more than 90 days before their first appointment or no more than 30 days after the first appointment. For six-month values, we used the first value 182 days or more after the baseline visit. In those instances where there were no available values at six-months or more, we used their last value as long as it was 45 days or more after the baseline value. The only exception was for HbA1c, where we increased the inclusion criteria to at least 60 days after the first appointment.

Lastly, we calculated the percentage of patients with clinically meaningful improvement in clinical outcomes between baseline and six-months for the full sample and then within diagnoses for clinically relevant outcomes. These data are shown for descriptive purposes only. We did not assess for clinically meaningful changes in HbA1c for the full sample because two-thirds of patients did not have type 2 diabetes; thus, their HbA1c levels were more likely to have been in “control” at baseline and any clinically meaningful improvement detected would not be representative of the full sample.

The sample size in analyses fluctuates due to missing outcome data. We used Wilcoxon signed-rank tests to assess changes in means of the clinical outcomes. This test is the non-parametric test used when data are not normally distributed. When sample sizes were less than 20, we did not perform statistical tests in order to avoid comparing estimates with limited statistical stability. In those cases, we present data descriptively. All analyses were conducted using SPSS version 26 (42). Results were considered statistically significant if they had a value of *p* less than 0.05.

## Results

### Demographic characteristics, baseline diagnoses, and days between measures

Table 1 shows the demographic characteristics and baseline diagnoses of all PBLM Program patients, stratified by gender. The majority of patients were female (69.4%) and were, on average, about 55 years old. The majority of patients had a baseline diagnosis of overweight or obesity (87.3%), roughly half of the sample was hypertensive or had a diagnosis of hyperlipidemia (53.2 and 49.7%, respectively), about one-third had a baseline diagnosis of type 2 diabetes (33.5%), a little over one-fifth had prediabetes (21.4%), and a small percentage of the sample had a baseline diagnosis of heart disease (12.1%). There was a statistically significant difference in heart disease among males and females. Specifically, a larger percentage of male patients had a baseline diagnosis of heart disease as compared to female patients (22.6% vs. 7.5%). The average number of days between patients’ baseline and six-month measures varied: HbA1c 172 days (SD = 70), weight, systolic blood pressure, diastolic blood pressure 169 days (SD = 66); non-HDL-C 167 days (SD = 81); LDL-C 167 days (SD = 80) (not shown).

**Table 1:** Sample characteristics and baseline diagnoses by gender (*n* = 173).

Characteristic	Full sample		Males		Females	
	*N*	Mean (SD) or Percent	*N*	Mean (SD) or Percent	*N*	Mean (SD) or Percent
Gender	173					
Male		30.6	53	100.0		
Female		69.4			120	100.0
Age	173	55.0 (12.0)	53	54.2 (11.5)	120	55.3 (12.3)
Overweight/Obesity	173		53		120	
No		12.7		17.0		10.8
Yes		87.3		83.0		89.2
Type 2 Diabetes	173		53		120	
No		66.5		58.5		70.0
Yes		33.5		41.5		30.0
Prediabetes	173		53		120	
No		78.6		84.9		75.8
Yes		21.4		15.1		24.2
Hypertension	173		53		120	
No		46.8		47.2		46.7
Yes		53.2		52.8		53.3
Heart Disease	173		53		120	
No		87.9		77.4		92.5*
Yes		12.1		22.6		7.5
Hyperlipidemia	173		53		120	
No		50.3		50.9		50.0
Yes		49.7		49.1		50.0

*p < 0.05.

### Average change in clinical outcomes over time

Table 2 displays findings for all six clinical outcomes for the full sample. We then stratified by baseline condition and show weight in addition to the measure most relevant to that condition. Among the full sample of patients, including those without overweight/obesity, type 2 diabetes, prediabetes, hypertension, or hyperlipidemia, there were statistically significant reductions in patients’ weight (92.5 kg vs. 88.8 kg), HbA1c levels (6.7% vs. 6.3%), and diastolic blood pressure (76.6 mm Hg vs. 73.9 mm Hg) over time. Change in LDL-C was approaching significance (101.7 mg/dl vs. 93.0 mg/dl, *p* < 0.10). For the subgroup of patients that had a baseline diagnosis of overweight or obesity (*n* = 151), we found significant improvements in weight (95.6 kg vs. 91.7 kg). For the subgroup of patients with type 2 diabetes (*n* = 58), there were improvements in HbA1c (7.9% vs. 7.2%) and weight (98.2 kg vs. 93.3 kg). For the subgroup of patients with prediabetes (*n* = 37), only weight was evaluated for statistical significance, as there were too few patients with HbA1c measurements at both timepoints to perform statistical tests. Weight, on average, decreased from 88.1 kg to 85.0 kg (*p* < 0.05) for those with prediabetes. Statistically significant improvements were found in diastolic blood pressure among patients with a diagnosis of hypertension (*n* = 92; 79.1 mm Hg vs. 75.0 mm Hg) and weight (92.0 kg vs. 88.5 kg) between baseline and six-months. No statistically significant changes were detected in systolic blood pressure. Lastly, among individuals with hyperlipidemia (*n* = 86), the change in LDL-C was approaching significance (112.1 mg/dL vs. 98.0 mg/dl, *p* < 0.10) and weight was significant (93.2 kg vs. 90.0 kg).

**Table 2:** Clinical outcomes for full sample and by baseline diagnosis.

	Full sample (*n* = 173)			Overweight/Obesity (*n* = 151)			Type 2 diabetes (*n* = 58)			Prediabetes (*n* = 37)			Hypertension (*n* = 92)			Hyperlipidemia (*n* = 86)		
		Baseline	Six-month		Baseline	Six-month		Baseline	Six-month		Baseline	Six-month		Baseline	Six-month		Baseline	Six-month
	*N*	Mean change (SD)	Mean (SD)	*N*	Mean (SD)	Mean (SD)	*N*	Mean (SD)	Mean (SD)	*N*	Mean (SD)	Mean (SD)	*N*	Mean (SD)	Mean (SD)	*N*	Mean (SD)	Mean (SD)
Weight (kg)	113	92.5 (22.1)	88.8 (22.1)*	100	95.6 (21.4)	91.7 (21.7)*	37	98.2 (23.9)	93.3 (23.0)*	24	88.1 (13.2)	85.0 (12.7)*	57	92.0 (20.2)	88.5 (19.1)*	54	93.2 (24.7)	90.0 (24.8)*
HbA1c (%)^a^	75	6.7 (1.7)	6.3 (1.2)*				32	7.9 (1.7)	7.2 (1.1)*	17	5.8 (0.2)	5.8 (0.3)^a^						
Systolic blood pressure (mm Hg)	115	126.1 (19.5)	123.0 (16.0)										60	133.8 (21.6)	128.8 (16.2)			
Diastolic blood pressure (mm Hg)	115	76.6 (10.7)	73.9 (9.8)*										60	79.1 (11.1)	75.0 (10.3)*			
Non-HDL-C (mg/dL)	70	130.8 (44.1)	121.3 (36.6)													38	140.3 (45.8)	125.3 (38.9)
LDL-C cholesterol (mg/dL)	70	101.7 (38.9)	93.0 (34.7)^†^													38	112.1 (42.2)	98.0 (38.9)^†^

Ns may vary due to missing data. The subgroups of patients by baseline diagnoses are not mutually exclusive. ^a^A statistical test assessing change in HbA1c over time was not performed for the subgroup of patients with a baseline diagnosis of prediabetes due to the small sample size. ^†^*p* < 0.10; *p < 0.05.

### Average change in clinical outcomes for those with a stable medication regimen

We assessed changes in clinical outcomes for patients with a baseline diagnosis of type 2 diabetes, hypertension, or hyperlipidemia who also had a stable or reduced disease-specific medication regimen (Table 3). Among those with type 2 diabetes, there were statistically significant improvements in HbA1c (7.8% vs. 7.1%) and weight (97.2 kg vs. 91.9 kg). For those with hypertension, we detected a significant reduction in diastolic blood pressure from 79.8 mm Hg to 75.4 mm Hg over time in addition to weight (92.0 kg vs. 88.6 kg). Changes in both non-HDL-C and LDL-C approached statistical significance (140.4 to 124.7 and 112.8 to 98.6 mg/dl, respectively) between baseline and the six-month visit for those with hyperlipidemia while there was a statistically significant improvement in weight (92.9 kg vs. 89.7 kg).

**Table 3:** Clinical outcomes by stable or reduced medication regimen for patients with a baseline diagnosis of type 2 diabetes, hypertension, or hyperlipidemia.

	Stable or reduced diabetes medication regimen (*n* = 36)			Stable or reduced blood pressure medication regimen (*n* = 55)			Stable or reduced cholesterol medication regimen (*n* = 55)		
		Baseline	Six-month		Baseline	Six-month		Baseline	Six-month
	*N*	Mean (SD)	Mean (SD)	*N*	Mean (SD)	Mean (SD)	*N*	Mean (SD)	Mean (SD)
Weight (kg)	28	97.2 (23.3)	91.9 (22.5)*	47	92.0 (18.4)	88.6 (17.7)*	47	92.9 (24.9)	89.7 (25.1)*
HbA1c (%)	26	7.8 (1.5)	7.1 (1.2)*						
Systolic blood pressure (mm Hg)				48	133.6 (21.9)	128.3 (15.5)			
Diastolic blood pressure (mm Hg)				48	79.8 (10.9)	75.4 (9.6)*			
Non-HDL-C (mg/dL)							34	140.4 (48.3)	124.7 (39.0)^†^
LDL-C (mg/dL)							34	112.8 (44.1)	98.6 (40.0)^†^

Ns may vary due to missing data. The subgroups of patients by stable medication regimen are not mutually exclusive. ^†^*p* < 0.10; *p < 0.05.

### Clinically meaningful change in outcomes over time

Table 4 shows the proportion of patients who had clinically meaningful change over time for the full sample and then by baseline diagnosis. In the full sample, more than half of patients had clinically meaningful changes in four of the five outcomes assessed (HbA1c was not assessed for the full sample). Specifically, a substantial proportion of patients had improvements in their weight (50.4%), diastolic blood pressure (50.4%), non-HDL-C (54.3%), and LDL-C (55.7%); this includes patients who did not have a baseline diagnosis of hypertension or hyperlipidemia. One half of those with overweight or obesity were found to have a clinically meaningful reduction in weight. For those with type 2 diabetes, we determined that at least half of patients had clinically meaningful improvements in HbA1c (50.0%) and weight (51.4%). Among those with prediabetes at baseline, a sizable proportion had clinically meaningful outcomes in both HbA1c (76.5%) and weight (50.0%). Forty percent of those with hypertension at baseline had a clinically meaningful reduction in systolic blood pressure, 56.7% had improvement in diastolic blood pressure, and 56.1% had a clinically meaningful reduction in weight. Additionally, almost two thirds of the hyperlipidemia subgroup (60.5%) had clinically meaningful reductions in both non-HDL-C and LDL-C over time, while 51.9% had a meaningful improvement in weight.

**Table 4:** Percent of patients with a clinically meaningful improvement in clinical outcomes for full sample and by baseline diagnosis.

	Full sample (*n* = 173)		Overweight/Obesity (*n* = 151)		Type 2 diabetes (*n* = 58)		Prediabetes (*n* = 37)		Hypertension (*n* = 92)		Hyperlipidemia (*n* = 86)	
	*N*	Percent	*N*	Percent	*N*	Percent	*N*	Percent	*N*	Percent	*N*	Percent
Weight (kg)	113	50.4	100	50.0	37	51.4	24	50.0	57	56.1	54	51.9
HbA1c (%)^a^	75	n/a			32	50.0	17	76.5				
Systolic blood pressure (mm Hg)	115	42.6							60	40.0		
Diastolic blood pressure (mm Hg)	115	50.4							60	56.7		
Non-HDL-C (mg/dL)	70	54.3									38	60.5
LDL-C (mg/dL)	70	55.7									38	60.5

Ns may vary due to missing data. The subgroups of patients by baseline diagnoses are not mutually exclusive. Clinically meaningful improvement in clinical outcome was defined as follows: Weight: a minimum 3% decrease in total body weight. HbA1c for those with type 2 diabetes: a minimum 0.5 percentage point reduction. HbA1c for those with prediabetes: no change or any decrease in HbA1c, signifying lack of progression to type 2 diabetes. Systolic and diastolic blood pressure: a minimum 3 mm Hg reduction in systolic and diastolic blood pressure. Non-HDL-C and LDL-C: any decrease. ^a^The percent of patients with a clinically meaningful improvement in HbA1c is not shown for the full sample because nearly half of the patients in the full sample did not have prediabetes or diabetes.

## Discussion

We have previously published findings that the pilot PBLM Program is feasible with high levels of patient satisfaction and interest (31). In this study, we now demonstrate a significant reduction in key biomarkers of cardiometabolic disease, including HbA1c, body weight, lipids, and diastolic blood pressure. We report findings that are both statistically significant and represent clinically meaningful improvements related to cardiometabolic risk. While some residential, community-based, employee health, and/or pay-out-of-pocket lifestyle programs have also shown improvement in biomarkers (43–45), this study demonstrates that a lifestyle medicine program based in the primary care clinic of a resource-limited safety-net hospital, working within traditional administrative and insurance structures, can be effectively delivered to patients while also achieving clinically meaningful improvements.

The goal of identifying patients with prediabetes is to help implement healthy lifestyle behaviors to prevent the progression to type 2 diabetes and its complications. Of those identified with prediabetes in our program, 76.5% successfully reduced their HbA1c or avoided an increase. Long-term follow-up of combined diet and exercise programs has demonstrated success in preventing diabetes while also achieving cost savings (46, 47). Among the patients with type 2 diabetes, the average HbA1c reduction was 0.7 percentage points, on par with many glucose-lowering medications (48). Moreover, 50% were able to attain at least a 0.5% reduction in their HbA1c, an amount that has historically been considered clinically meaningful, especially since a positive dose–response association has been reported between HbA1c and cardiovascular outcomes and mortality (34, 35). A meta-analysis by Giugliano et al. evaluated cardiovascular outcome trials of diabetes medications and demonstrated that a 0.5% reduction in HbA1c was associated with a 20% reduction in major cardiovascular events (MACE) (36). Clinically meaningful HbA1c lowering was reported only for patients with diabetes or prediabetes, as further lowering of blood sugar among patients with normal HbA1c is less likely to occur and is of uncertain clinical benefit.

Half of the patients lost at least 3% of their body weight, an amount that is clinically meaningful as it has been shown to translate into meaningful improvement in obesity-related comorbidities (33). Notably, the weight loss occurred without prescribed calorie restriction. The program emphasizes the consumption of unprocessed or minimally processed plant-foods – which are high in fiber and nutrient density, and low in energy density – while encouraging patients to limit or avoid animal products, saturated fats, sodium, added sugars, and ultra-processed foods. Several factors may contribute to the weight-management benefits of a healthful plant-based diet: higher fiber, lower energy density, gut microbiome effects, and increased thermogenesis (49).

We used a 3-mm Hg drop in either systolic or diastolic blood pressure as clinically meaningful as this aligns with the FDA’s criteria for approval of blood pressure lowering medications (38, 39). Moreover, evidence suggests that even modest reductions in blood pressure have the potential to result in meaningful decreases in cardiovascular risk (50, 51). In the entire study population, including those without a diagnosis of hypertension, 43% of patients achieved at least a 3-mm drop in systolic blood pressure and 50% achieved at least a 3-mm drop in diastolic blood pressure. Among those with a diagnosis of hypertension, 40% of patients attained this clinically meaningful drop in systolic blood pressure and 57% attained this clinically meaningful drop in diastolic blood pressure, signifying an important reduction in cardiovascular risk (52, 53).

The baseline LDL-C of patients with hyperlipidemia in our pilot program was 112 mg/dl, with the majority already on statin therapy to lower cholesterol. Long-term drug treatment studies have shown that there is a continuous relationship between LDL-C and MACE, even at low levels of LDL-C (41, 54). For this reason, any LDL-C lowering appears to be clinically meaningful. In this pilot program, 60.5% of patients with hyperlipidemia experienced a decrease in their LDL-C and non-HDL-C. Among those on a stable or reduced cholesterol medication regimen, the average LDL-C was lowered by 14.2 mg/dl to 98.6 mg/dl.

A key finding in this study is that clinical improvements occurred among patients with stable or reduced medication regimens for diabetes, blood pressure, or cholesterol. This strongly suggests that the noted improvements in glycemic control, diastolic blood pressure, lipids, and body weight were due to lifestyle changes themselves, rather than to increases in medications. Typically, healthy lifestyle behaviors are synergistic with medications, resulting in clinical improvements and lower cardiometabolic risk without higher medication burden (21–25). In some cases lifestyle changes may allow for the reduction or cessation of medication. These benefits extend not only to patients’ short- and long-term health, but to lower cost of prescription medications and lower risk of medication side effects.

There are limitations to this study worth noting. First, the majority of patients of the PBLM Program were highly motivated individuals who self-referred into the program. Consequently, the findings may be biased upwards if these individuals were more earnest in their attempts to adhere to program recommendations than a typical patient. Second, some patients reported they were already following a healthful plant-based diet at baseline. Therefore, it is also possible that the study did not fully capture the clinical benefits of transitioning from a less-healthful dietary pattern; this would bias the findings toward the null. Third, findings for the full sample includes patients with a variety of baseline conditions potentially obscuring changes for individual outcomes when only a subset of patients would be expected to improve. Fourth, without a comparison group, we cannot be certain that improvements in clinical outcomes were a result of participation in the PBLM Program. Fifth, certain clinical measurements used in this study may be unreliable. For example, in-office blood pressure measurements are known to be unreliable due to “white-coat” effects and improper measurement technique (55, 56). Additionally, lipid measurements may have been impacted by inconsistency in whether patients fasted prior to measurement. However, this limitation does not apply to non-HDL-C measurements which are not significantly affected fasting status. Sixth, it is also important to note that at times sample sizes were rather small, particularly when evaluating subgroups. Small sample sizes result in reduced power to detect statistically significant differences, as well as increased margin of error. However, because the primary goal of the evaluation was to assess implementation and demand, this study was not designed or powered to assess the PBLM Program’s impact on clinical outcomes and can only provide a preliminary signal as to the effectiveness of the program. Despite these limitations, this study is novel and to our knowledge, the first of its kind to include an under-resourced and vulnerable population in a public healthcare setting.

Given the burden of chronic disease and the high cost of health care, it is essential that the care delivery system move beyond merely managing lifestyle-related illnesses, but rather, addressing their key root causes. Lifestyle medicine, where providers help build a foundation for healthful eating, active living, and emotional resilience, is a promising approach for reaching this goal. The findings from this pilot study are encouraging and may have implications for healthcare practitioners and health systems. Further study is needed to demonstrate additional health benefits over time and the potential for cost-savings to society.

## Data availability statement

The raw data supporting the conclusions of this article will be made available by the authors, without undue reservation.

## Ethics statement

Approval for this study was obtained by the NYU Grossman School of Medicine Institutional Review Board and the Office of Research and Administration for Implementation at NYC Health + Hospitals/Bellevue. Written informed consent was not required for this study in accordance with the national legislation and the institutional requirements.

## Author contributions

SLA and MM co-designed the study. SLA led the study and drafted the manuscript. REM led data management and performed data analysis with LK. LC and MM led data acquisition. HEA advised data analyses. All authors contributed to the interpretation of the results, writing the manuscript, and approved the final draft.

## Funding

This work was supported by NYC Health + Hospitals.

## Conflict of interest

The authors declare that the research was conducted in the absence of any commercial or financial relationships that could be construed as a potential conflict of interest.

## Publisher’s note

All claims expressed in this article are solely those of the authors and do not necessarily represent those of their affiliated organizations, or those of the publisher, the editors and the reviewers. Any product that may be evaluated in this article, or claim that may be made by its manufacturer, is not guaranteed or endorsed by the publisher.
